# Game theoretical approach for load balancing using SGMLB model in cloud environment

**DOI:** 10.1371/journal.pone.0231708

**Published:** 2020-04-20

**Authors:** R. Swathy, B. Vinayagasundaram, G. Rajesh, Anand Nayyar, Mohamed Abouhawwash, Mohamed Abu Elsoud

**Affiliations:** 1 Computer Center, MIT Campus, Anna University, Chennai, Tamil Nadu, India; 2 Department of Information Technology MIT Campus, Anna University, Chennai, Tamil Nadu, India; 3 Graduate School, Duy Tan University, Da Nang, Vietnam; 4 Department of Mathematics, Faculty of Science, Mansoura University, Mansoura, Egypt; 5 Department of Electrical and Computer Engineering, Michigan State University, East Lansing, MI, United States of America; 6 Computer Science Department, Faculty of Computers and Information, Mansoura University, Mansoura, Egypt; 7 Computer Science Department, University of Tabuk, Tabuk, Saudi Arabia; Shandong University of Science and Technology, CHINA

## Abstract

On-demand cloud computing is one of the rapidly evolving technologies that is being widely used in the industries now. With the increase in IoT devices and real-time business analytics requirements, enterprises that ought to scale up and scale down their services have started coming towards on-demand cloud computing service providers. In a cloud data center, a high volume of continuous incoming task requests to physical hosts makes an imbalance in the cloud data center load. Most existing works balance the load by optimizing the algorithm in selecting the optimal host and achieves instantaneous load balancing but with execution inefficiency for tasks when carried out in the long run. Considering the long-term perspective of load balancing, the research paper proposes Stackelberg (leader-follower) game-theoretical model reinforced with the satisfaction factor for selecting the optimal physical host for deploying the tasks arriving at the data center in a balanced way. Stackelberg Game Theoretical Model for Load Balancing (SGMLB) algorithm deploys the tasks on the host in the data center by considering the utilization factor of every individual host, which helps in achieving high resource utilization on an average of 60%. Experimental results show that the Stackelberg equilibrium incorporated with a satisfaction index has been very useful in balancing the loading across the cluster by choosing the optimal hosts. The results show better execution efficiency in terms of the reduced number of task failures by 47%, decreased ‘makespan’ value by 17%, increased throughput by 6%, and a decreased front-end error rate as compared to the traditional random allocation algorithms and flow-shop scheduling algorithm.

## Introduction

Cloud Computing platform consists of several remote servers that provide a large number of cloud-based services like Infrastructure Service, Platform Service, and Software Service [1–3]. These platforms provide storage and computing capabilities as a subscription-based service in the pay-as-you-go model. Cloud data center provides storage and computing facilities on the internet through a large number of physical hosts. Each physical host has resources like CPU, memory to provide service to the users. The available resource of the physical host changes continuously based on the tasks submitted to the host. The performance of the data center depends on the tasks deployed on the host. If the host in the data center has a sufficient amount of resources to deploy the tasks submitted, the performance of the host will be high that it can execute the task successfully and respond with the result on time to the user. The host with an insufficient resource cannot deploy the task resulting in performance degradation and failure of the task. The challenge lies in allocating appropriate tasks to optimal hosts in a load-balanced way that improves the performance of the data center, thus decreases the failure number of tasks.

Load balancing in the cloud environment is an important issue to be considered in recent days. Load balancing is a method to distribute the load evenly to the entire host in the cloud dynamically. There are many methods designed to support load balancing in the ‘Local Area Network,’ but very few strategies exist to perform load balancing in the cloud environment. Load balancing based on task scheduling, probabilistic approach, and other optimized algorithms exist to improve the load balancing metrics. The various performance metrics considered for load balancing are makespan, throughput, latency, response time, resource utilization, degree of imbalance and cost [4, 5]. Various methods perform load balancing, which is suitable for a short duration. A novel game-theoretical based approach has been proposed for load balancing task which performs well in the long run.

Game theory is a mathematical model that can be used for decision making among firms that sell different products. The firms are treated as the players of the game. In a game, one of the players makes their first move and the other players make the next move based on the first player. Thus, the decision of a player influences the outcome model of the other player. In Game theory, each player makes a move by choosing their best strategy such that every player in the game is benefitted. Game theory is modeled by the players, strategies, outcomes, and payoff. The players are the decision makers, strategies are the course of action that a player may choose to follow, outcome is the results based on the strategy chosen by the player, and finally, payoff is the cost that the players receive for a particular outcome. There exist different categories of the game. First, the game is categorized based on the number of players as one player game, two-player games, or n-player games. Second, based on the rationality of the player, the game is categorized in two ways as one in which the players make an intelligent or rational move while in other the players make the random move. Third, is cooperative game in which players make their decisions jointly and non-cooperative game in which the players make their individual move. Finally, we have zero-sum and non-zero-sum games in which the sum of payoffs of all the players is equal to zero. Equilibrium is a concept of game theory where each player in the game arrives at their optimal outcome. Considering the advantage, the mathematical model based on game theory strategy has been utilized for balancing tasks load among physical hosts in Cloud Data Center such that every host and users are benefitted. The proposed model is independent of the cloud models and can be applied on public, private, and hybrid clouds as long as enough security controls relevant for the respective cloud models are implemented. Among the various game-theoretical approaches available, we use the Stackelberg Game theoretical Model for task load balancing.

Stackelberg Model is a leadership model, i.e., it has a leader who chooses its best strategy based on which other followers choose their strategy to maximize their benefit. Stackelberg's model is also named as the leader-follower game. In this model, the decisions are made sequentially, i.e., the leader firm makes the first decision, and then the follower firm makes their next decision. Thus the leader firm gets the maximum profit by contributing the followers, which is defined as the Stackelberg equilibrium. Equilibrium is the state where the players have made their decision, and the desired outcome arrives. SGMLB—Stackelberg Game Model for Load Balancing algorithm has been provided to balance the task load among the physical host in the cloud data center. The Stackelberg leader-follower game model is a centralized load balancing strategy for task deployment in Cloud Data Center. Here the centralized approach is considered in contrast to the decentralized approach as synchronizing many nodes in a decentralized approach is a costly affair and may lead to suboptimal decisions. The cloud environment is simulated with ‘n’ number of hosts in a data center with a single load balancer to handle the incoming load to the data center. The load balancer receives the available load of the data center and the request load of the task. The load balancer executes the SGMLB algorithm and based on the follower’s strategy, the load balancer allocates the tasks to the host. The load balancer takes the role of a leader, and all other hosts behave like the follower.

The main objective of the proposed work is to deploy the tasks among cloud data centers in such a way that all the tasks are evenly distributed to the hosts with requested resources. No host in the data center is overloaded, and the available resources are efficiently utilized. All of this will contribute to performance efficiency and decrease the number of task failures. The proposed Game theoretical approach for load balancing with price strategy and satisfaction factor finds the suitable physical host with the right amount of resource and assigns tasks to that hosts in a cloud data center. This ensures that the task completes without any failures, and the resource in every physical host is productively utilized. The proposed work measures throughput, makespan, task failures, resource utilization, and front-end error rate using the SGMLB algorithm incorporated with a satisfaction index in a simulated environment using cloudsim.

The rest of the paper is organized as follows: In section 2, various existing works for load balancing in cloud computing environments are discussed. Section 3 presents the detailed problem formulation of the Stackelberg game-theoretical model for load balancing. Section 4 highlights the algorithm of proposed Model. Section 5 elaborates the mathematical derivation regarding how Stackelberg equilibrium is maintained. In Section 6 and 7, the simulation environment, metrics evaluation, and the comparison of the performance parameters are discussed. Section 8 concludes the paper with a discussion on how the SGMLB model allocates tasks to the physical host in the cloud environment along with the future scope.

## Related work

Various studies are proposed, and the number of researches is steadily increasing in recent times related to dynamic load balancing in the cloud data center to handle the computing resources efficiently. As the industries move towards Business 4.0, where automation and remote computing play a significant role, optimal load balancing has become the widely studied topic in recent times. Nayyar [6] provided all the essentials of Cloud Computing like cloud types, its features, cloud components, their advantages, and disadvantages. The concept of virtualization, services of the cloud, and cloud security also discussed in detail along with a detailed overview of all available simulation environments for the cloud. Centralized load balancing and distributed load balancing techniques are widely used load balancing techniques cloud data centers [3–8]. In centralized load balancing, the coordinator or the central node plays the primary role in allocating and de-allocating the resources. In distributed load balancing, multiple nodes act as the coordinator and do the load balancing job. VM (Virtual Machine) scheduling algorithms also exist to support load balancing. Researchers have proposed a series of scheduling methods like FCFS, Round Robin, least connection method, and load balancing methods like min-min, max-min, Honeybee, Ant-colony optimization to solve load balancing issues in real-time cloud environments [9, 10].

Farrag et al. [11] discussed load balancing algorithms for cloud environment like VM scheduler using Bayes theorem, Artificial Bee Colony Algorithm, and Genetic algorithm. The advantages of these algorithms are its ease of use, but when considered for cloud data centers, their load balancing effect is not absolute. Like other load balancing algorithms, Kaur et al. [12] discussed the data placement in cloud computing based on workflow management that has a direct impact on performance, cost, and execution time of workflows. The Workflow Management Coalition (WfMC) has been defined as an advancement in organizing exercises and calculations of a business procedure. WfMC introduces a reference framework model that empowers the process to operate interactively at an assortment of IT applications. The workflow lifecycle comprises of the workflow design phase, partitioning, mapping, and enactment. Then the data placement process is carried out as a movement of input data of a data-intensive application from a remote site to the execution site, and then the output is moved from the execution site to remote site or the same site. Various data placement methods have been discussed, and few are based on correlation, genetic algorithm, energy, PSO, ACO, replication, and fault tolerance. These algorithms were implemented in various cloud simulation tools, and the data placement criteria were analyzed. In contradiction with load balancing and data placement algorithms, Deep et al. [13] proposed an authentication mechanism for cloud databases using blockchain technology. A novel authentication algorithm was proposed by authenticating login credentials using the blockchain mechanism. Both insiders and outsiders are authenticated using IDs and signatures that secure the system from insiders and outsiders attack. The performance of the system is evaluated using the scyther tool.

Zhao et al. [14], deployed the tasks among physical hosts in the cloud data center by a probabilistic approach using Bayes Clustering (LB-BC) and discussed how the LB-BC model could achieve overall load balancing. The combination of Bayes theorem and clustering process has obtained the optimal clustering set of physical hosts on which the tasks were deployed. Wang et al. [15] proposed a SDN based dynamic load balancing in the cloud data center. The work detects the load traffic of the open flow switching network. From the load traffic, the load variance of the servers in the network is calculated, and the incoming load is hashed to the servers with the least load. The hashing technique for load distribution does not guarantee load balancing in the long run. Tang et al. [9], suggested load-balanced scheduling (DLBS) approach to analyze the load imbalance degree of every data center in the open flow network and then schedule the unbalanced data load to other data centers in order to maximize network throughput. Somula et al. [16], proposed load balancing in mobile computing to improvise response time and reduce waiting time and latency. Load balancing is carried out by offloading the load from mobile cloudlets to cloud computing environments using a round-robin algorithm having limited resources like storage, processing capacity, and battery life.

Patel et al. [17], introduced resource provisioning and allocation along with task scheduling in a distributed environment using priority-based round-robin method by allocating time slices for every task. Zhang et al. [18], performed network-aware virtual machine migration in the overloaded cloud and used the Artificial-Bee Colony optimization algorithm and Genetic algorithm to achieve load balancing effect. It was observed after experimentations that Artificial Bee Colony outperformed the Genetic algorithm in terms of data transfer time. However, the method results in low resource utilization. Sun et al. [19] proposed a load balancing technique using Flowshop scheduling to maintain fairness strategy in a parallel environment. This parallelization of Flowshop scheduling is done using the hierarchical master-worker paradigm. The task is distributed by the supervisor processes to master processes and then to the worker processes in hierarchical order. The nodes are allocated with different amounts of tasks based on their completion. The supervisor took the responsibility of the load balancer and distributed the tasks. This method faces communication overhead in the process, which affects system performance.

Game theory is a mathematical model that focuses on decision making between self-interested agents and is widely used for developing decision-making strategies for co-operation among rational decision-making entities as proposed by Wooldridge, Michael [20]. Each player in the entity uses their rational choice to make their best decision. The computational challenges in applying game theory concepts in AI were addressed where cooperative game theory is used. Game theory is interpreted as descriptive or normative interpretation. Descriptive interpretation foresees the behavior of players in a specific strategic setting, and normative interpretation imposes the action that a player needs to make. In recent times, the algorithmic game theory is a significant growth area in theoretical computer science. There are different game-theoretical approaches available like cooperative and non-cooperative games [21,22], symmetric and non-symmetric, simultaneous and sequential move games, constant sum, zero-sum and non-zero- sum games. Stackelberg model is one of the strategic game models in which the leader firm makes its first move, and then the follower firm makes its next move sequentially [23]. This model is framed based on the game theory, where a group of players follows a leader to compete for a quantity or resource. Load balancing for future internet was proposed by Song et al. [24] using the game-theoretical approach in modeling the static load balancing in which the game is modeled as a non-cooperative game among users considering minimal response time for the task and cooperative game among processors considering minimal system processing time.

Nan et al. [25], discussed the distribution of live multimedia streaming data from the cloud to both desktop and mobile users using a two-stage Stackelberg game: an evolutionary game model for mobile users and a non-cooperative game model for the desktop users. This approach allows a mobile user with limited bandwidth to acquire live multimedia streaming from desktop users. Their strategies comprise of the bandwidth size and price. The bandwidth related problem between desktop users and mobile users is solved using a non-cooperative game and evolutionary game model for sharing the bandwidth and price by maximizing their utilities. Yu and Hong [26], provided optimal load control of a device in the virtual electricity trading process based on demand response (DR) algorithm using real-time price. In this study, one leader, N-follower game, has been formulated in which the energy management center is the virtual retailer who acts as the leader and the other devices that purchase energy acts as the follower. The DR algorithm helps each device in selecting the optimal strategy for their energy demands based on a rapidly updating real-time price. The existence of Stackelberg equilibrium was also proved to state that both the leader and the followers are benefitted. This Stackelberg model helps to achieve load control of the device. Wang et al. [27], used mobile devices to share their unused resources for cooperative application execution for mobile cloud computing. To increase the benefit of the owners of the task and mobile devices in task execution, the Stackelberg game is formulated with the amount of processing unit that each mobile computing device can offer and at what price. The existence of Stackelberg equilibrium is shown using an efficient algorithm.

Duong et al. [28], performed power allocation in the cellular network by understanding the behavior of macrocell base station (MBS) and femtocell base stations (FBS), and modeled it as Stackelberg Bayesian Game. The cellular network consists of macrocells underlaid by the femtocell network in which MBS acts as the leader, and FBS acts as the follower. The MBS offers its price strategy for maximizing its overall benefit based on which the followers decide to maximize the transmission capacity of the femtocell network. It is taken care that the interference of the macro base station does not exceed an interference constraint, and the existence of Nash Equilibrium was also examined using backward induction. Tran et al. [29], used a two-stage Stackelberg game and modeled in the geo-distributed data center to distribute their substantial energy demands based on the real-time pricing scheme of demand response programs [30,23].

Based on the literature study, it is found there are a variety of methods for load balancing tasks, and in all those methods, load balancing has been done in an on-premise environment. Also, the Game theoretical approach has been used for solving various problems like bandwidth allocation in mobile computing, allocation of electrical energy on demand. In this research work, as an alternative to all the other methods, the Game theoretical approach has been used to load balance tasks in the cloud computing environment. For effective testing and implementation, metrics like makespan, throughput, and resource utilization have been analyzed. In addition to all the above metrics, the count of failed tasks and the front-end error rate has been measured.

The paper proposes SGMLB Game theoretical approach to efficiently perform load balancing for benefitting both the user’s tasks and the physical hosts of the cloud data center. In summary, the proposed work has the following aspects:

- Uses Stackelberg game theoretical model for load balancing task in cloud data center.
- Introduced Satisfaction factor in aggregating the favorable hosts for processing
- Utility function along with price strategy of cloud data center and satisfaction factor has been considered in allocating tasks to the physical hosts in data center. The price strategy of every physical host depends on its available CPU and memory resource.
- Tasks have been allocated to appropriate physical hosts with right amount of resources at best price and resources in data centre are efficiently utilized.

## Problem formulation

### Proposed work

**Fig 1:**
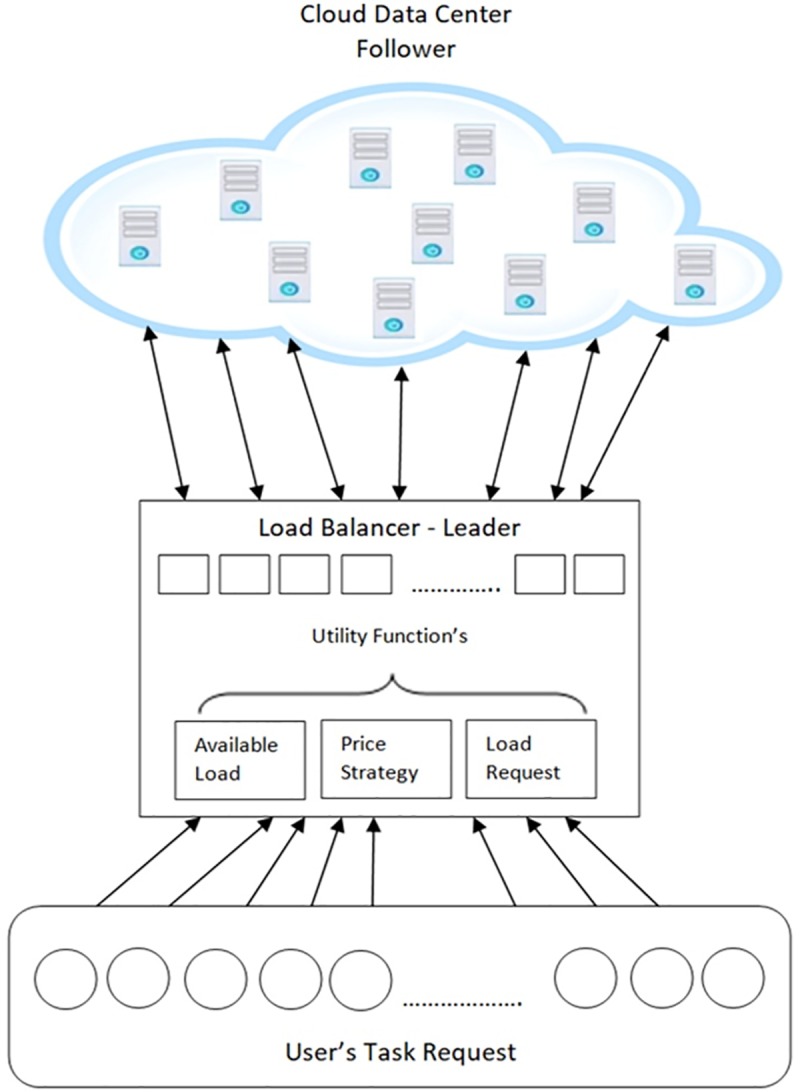
Stackelberg load balancer’s architecture diagram.

In cloud computing, where infrastructure is offered as a service (IaaS), clients consuming the service expect their tasks to be scheduled effectively with optimal pricing. When the tasks are submitted by the users, they are deployed on the physical hosts of the cloud data center. Generally, the tasks are deployed on the physical hosts randomly. When the random deployment of tasks is done, the task may get allocated to the host with inadequate resources compared to the resource requested by the tasks. This usually results in a delay in processing and more frequent failures in processing the incoming tasks. Also, if the task gets assigned to the host where the available computing resource is almost the same as the resource requested by the clients, it causes the host to be overloaded and results in service inefficiency [10]. Hence it is necessary to design an optimal model for task deployment in-order to balance the load in the cloud computing environment. The proposed solution to the problem deploys the task to a different host in the cloud data center based on Stackelberg game theoretical model. The architecture diagram of Stackelberg Load Balancer is given is highlighted in (Fig 1). The load balancer gets the load request from the user and receives the available computing resources of all the hosts in the cloud data center. Based on the available computing resources, the price is chosen from a range of values calculated based on the price strategy for the Data Center. With the available computing capacity, price strategy, and requested load, the utility function for the Data Center is calculated and the tasks are allocated to the optimal hosts with minimum utility value.

### Stackelberg game model based load balancing

The problem is formulated as follows: Consider the cloud data center with ‘m’ number of task requests arriving at time ‘t’ from the user. There are ‘n’ numbers of physical hosts in the Cloud Data Center to satisfy the user requirement. One of the nodes in the cloud data center is identified as the primary load balancer and another node as the stand-by load balancer, and the primary load balancer deploys the tasks to different hosts in the cloud based on Stackelberg game model. Stackelberg game model is a leadership model. The problem is applied to scenarios where the setup has one leader and ‘n’ followers. Here, the chosen load balancer in the cloud data center acts as the leader, and all other hosts act as followers. Throughout the paper, the term leader–load balancer and follower–hosts are used interchangeably. The work assumes that the cluster has high availability (HA) feature already implemented so that the cluster can fall back to the stand-by load balancer node and continue processing jobs if the primary node balancer becomes unresponsive after a certain period that is configured as per the HA parameters. The load balancer receives the overall storage and computation cost from the Cloud Service Providers (CSP) and also announces the available remaining resource amount of the physical host in the cloud periodically. The remaining resource amount includes the remaining CPU resource and the remaining memory resource. The leader receives m number of tasks requests from the users for processing, and they will have to be deployed to the available hosts in the cloud. We propose a novel approach of load balancing in task deployment strategy. The leader deploys the tasks request one by one to the physical hosts in a reliable manner, and this process occurs sequentially based on the Stackelberg Game Model for Load Balancing (SGMLB).

### Load balancer—Leader

All physical host in Cloud Data Center announces its available resource amount to the load balancer. The remaining resource amount includes the available CPU for processing and available memory for storage. The load balancer has a set (HS) of available CPU and memory of every individual host. Let HS = {(C^1^, M^1^), (C^2^, M^2^), (C^3^, M^3^), . . . .. . . .. . . .. . . ., (C^N^, M^N^)}. The set HS contains some unfavorable hosts which do not have sufficient resource amount to process the tasks request. The host with very substantial CPU resources and minimal memory resources or with extensive memory resources and minimal CPU resources are considered as an unfavorable host for processing. The load balancer eliminates those hosts by calculating the mean for the available CPU L_c_ and memory resource L_m_ using Eq (1)

$$L_{c}=\frac{\sum_{i=1}^{n}C^{i}}{n}and\;L_{M}=\frac{\sum_{i=1}^{n}M^{i}}{n}$$ (1)

Using the mean calculated, the host outliers are eliminated, thus forming a new host set HS’. From the new set formed, the load balancer calculates the total resource available for each host using the Eq (2).

$$L_{H}^{i}=\alpha C^{i}+\beta M^{i}\mathrm{where}\;\alpha +\beta =1$$ (2)

Here C^i^ is the remaining CPU resource, and M^i^ is the remaining memory of the host “I” in the cloud; α and β are the weight values of CPU and storage, which is determined by neural network learning. There arrives ‘m’ number of task requests at time ‘t’ from the users. Every task request requires C^j^ amount of CPU resource and M^j^ amount of memory resource. Based on the individual demand of the tasks the load balancer calculates the total load requirement of every task as given in Eq (3).

$$R_{T}^{j}=\alpha C^{j}+\beta M^{i}where\;\alpha +\beta =1$$ (3)

The price range for the cloud data center is fixed by the cloud service provider. The load balancer assigns the node for processing tasks based on its leader's strategy. Let p’ = {*p*_*1*_, *p*_*2*_, *p*_*3*_, *…*.., *p*_*N*_} be the price range based on the price strategy of the physical host or the followers. As per the Demand-Response scheme [30], the maximum price is chosen for the host with minimum computing resources available, and the minimum price is chosen from the price range for the host with maximum computing resources available. The tasks may be deployed on the physical host based on the follower’s strategy. The follower strategy consists of two parameters i.e. processing cost and the satisfaction factor.

- Processing cost: Given by the amount of load to be processed at price p_i_ and it is denoted as R_j_P_i._
- Satisfaction factor: Evaluates if the host can process the load request or not based on its current available CPU and memory.

The satisfaction factor is framed as an exponential function, which is a continuously increasing or decreasing function based on the resource demand R_j_. The satisfaction factor of the follower or the host is given in Eq (4).

SF=[e(1−RjLi)]−1 (4)

The satisfaction factor changes from negative to positive based on the request demand (R_j_). The satisfaction factor is expressed as an exponential function, which is a non-decreasing positive range function. The satisfaction factor is made to change between positive and negative values by adding -1 to the exponential function. If R_j_ is higher than L_i_, then the satisfaction factor would be negative, implying that the available load is less than the requested load. Hence the host cannot process the task request. If R_j_ value is less than the L_i_, then the satisfaction factor would be decisive, implying that the available load is higher than the task requested. So, the host can process the task requested. If R_j_ is equal to L_i_, the satisfaction factor would be zero implies that the load demanded and the load available are both the same. When the task gets assigned to such host, then the host may be overloaded and causes performance degradation. The tasks may be allotted to particular host only if the satisfaction factor of the host is positive. Thus, the feasible resource request that a host can process must be within an interval [$R_{j}^{min},R_{j}^{max}$] for a price in p’. The other parameter which plays the main role in task allocation to the host is the processing cost. Thus, the task gets allotted to a particular host whose utility function value of the follower is minimum and thus benefitting the users and the host. The hosts are benefitted in the way that appropriate task load gets allotted to the host so the hosts resources are efficiently utilized. The utility function for the follower is given in Eq (5).

$${UF}_{H}(p',R_{j})=p_{i}.R_{j}+w.\mathrm{SF}(R_{j}),w>0$$ (5)

The utility function value is computed for those hosts for which the satisfaction factor is positive. The negative or zero satisfaction factors implies that the hosts are unfavorable for processing the task requests. The price p_i_ for processing the task is calculated based on the available load. Thus, the price is decided based on a demand-response strategy where the maximum price is used when the available load is minimum and the minimum price is used in case on maximum available load. This price strategy helps to utilize the hosts with the maximum available load. The maximum and minimum prices are chosen within the price range of the cloud service provider. The optimal host for processing the user task is formulated as elaborated in Eq (6).

$$min{UF}_{H}(p',R_{j})$$ (6)

The utility function of the Leader or the load balancer is formulated as the net benefit obtained in processing the task for the given price and offloading the tasks to appropriate host in load-balanced d way. Thus, the utility functions is given in Eq (7).

$${UF}_{LB}(p',R\prime )=\sum_{i=1}^{n}p_{i}.R_{j}‐\sum_{i=1}^{n}w.\mathrm{SF}(R_{j}),w>0$$ (7)

The net benefit of the load balancer is computed based on the follower’s strategy. Utility value is calculated using the allotted tasks to the hosts as the summation of processing the request *R*_*j*_ at price *p*_*i*_ and the satisfaction factor of the request *R*_*j*_ in transferring to the hosts. The net benefit of the leader is maximized, thus indicating that almost all the tasks are assigned to appropriate hosts. The remaining resource amount is updated. Based on the maximum utilization of the hosts and based on the remaining resource amount available, the price of each host is chosen. On the whole, the overall performance of the cloud data center is improved. The optimal strategy of the host is illustrated in Eq (8).

$$max{UF}_{LB}(p',R\prime )$$ (8)

### Hosts–Follower

**Fig 2:**
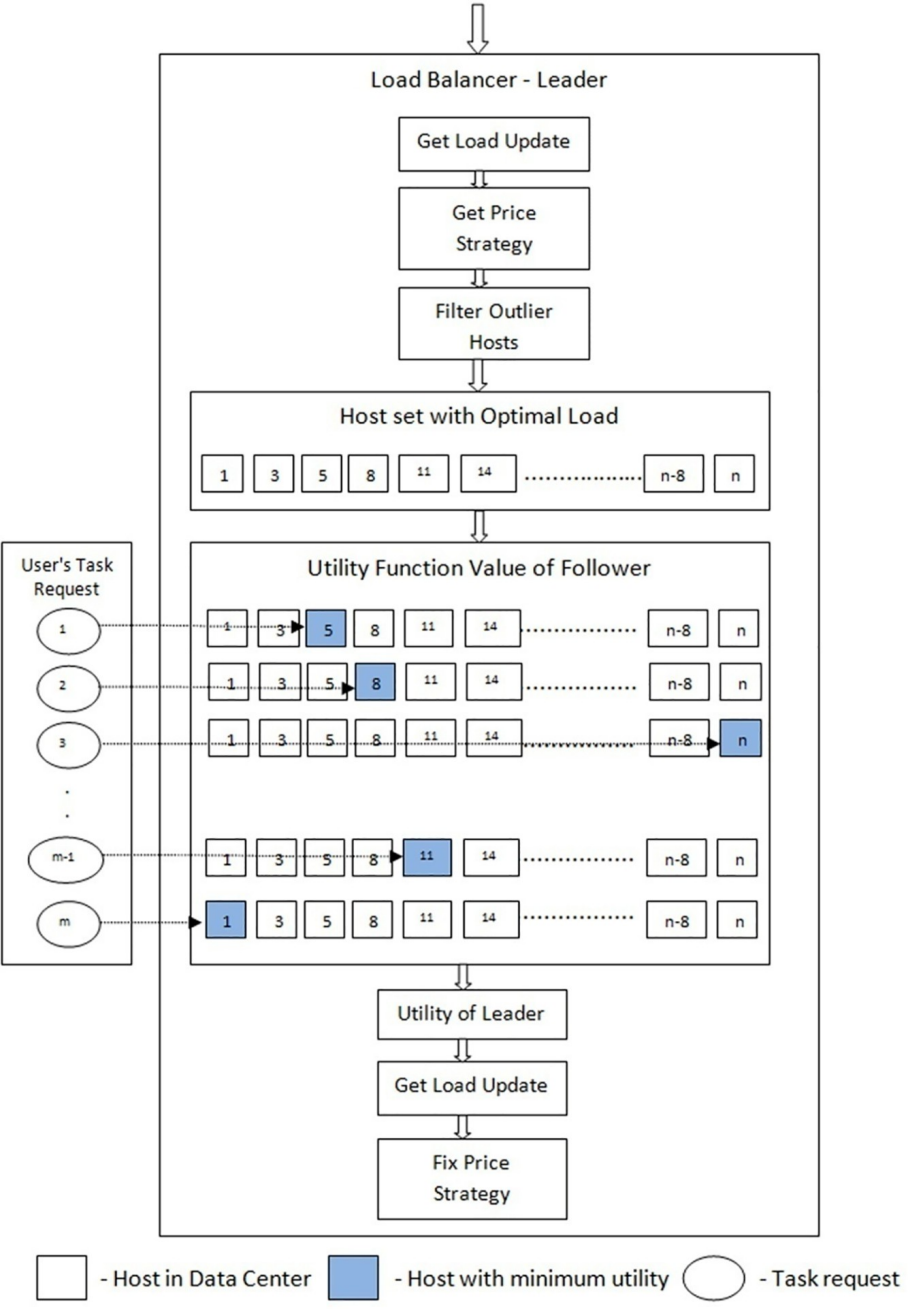
Flow diagram of SGMLB.

The hosts, i.e., the follower, chooses its best response strategy by selecting the minimum utility function value. Based on the best strategy, the follower accepts the task request and executes the same. The SGMLB algorithm flow is given in (Fig 2).

### Stackelberg game model

Stackelberg Game model has the following sequence of steps for performing task deployment:

1. The leader chooses the prize of every individual host based on the remaining resource amount and calculates the *UF*_*H*_(p',*R*_*j*_) for a host and incoming task requests and announces the utility strategy value to the follower.
2. The follower chooses *minUF*_*H*_(p',*R*_*j*_) as its best response strategy, thus utilizing its available resources efficiently.
3. Based on the best response strategy chosen by the follower, the leader identifies its optimal strategy as *maxUF*_*LB*_(p',*R*′) and update the price for every host based on the remaining resource amount.

Steps 1 to 3 are repeated at a regular time interval and as long as the task request arrives from the user.

Algorithm: SGMLB (H, C^i^, M^i^, TR, C^j^, M^j^)

        **Input. a**vailable H, available CPU C^i^, available memory M^i^, requested TR, requested CPU C^j^, requested memory M^j^

        **Output.** Load balancer (Leader) optimal strategy, Host (Follower) optimal Strategy, Price strategy for each host

    1. HS' = Φ;

    2. Chosen price strategy for each host p’ = {*p*_*1*_, *p*_*2*_, *p*_*3*_, *…*.., *p*_*N*_]

    3. Compute $L_{c}=\frac{\sum_{i=1}^{n}C^{i}}{n}$ And $L_{M}=\frac{\sum_{i=1}^{n}M^{i}}{n}$;

    4. if C^i^> L_C_ and M^i^> L_M then_

    5.    Add {(C^i^, M^i^) } to HS';

    6. End if

    7. for each hostH^i^ Є HS' do

    8.    L_H_^i^ = α C^i^ + β M^i^;

    9. End for

    10. for each task R^j^ Є TR do

    11.            R_T_^j^ = α C^j^ + β M^j^;

    12. End for

    13. for each hostH^i^ Є HS' do

    14.                    for each task R^j^ Є TR do

    15.                                        Compute satisfaction factor as SF=[e(1−RjLi)] - 1

    16.                                        if (SF> 0)

    17.                                                            *UF*_*H*_(p',*R*_*j*_) = *p*_*i*_.*R*_*j*_+w.SF(*R*_*j*_);

    18.                                        End if

    19.                    End for

    20. End for

    21. for each H^i^ Є HS' do

    22.                    Find *minUF*_*H*_(p',*R*_*j*_)as the optimal strategy for host H^i^ and assign task R^j^ to H^i^ with *minUF*_*H*_ and make host H^i^unavailable.

    23. End for

    24. Compute *UF*_*LB*_(p',*R*′) = $\sum_{i=1}^{n}p_{i}.R_{j}–\sum_{i=1}^{n}w.\mathrm{SF}(R_{j})$ for the load balancer

    25. The optimal strategy of the load balancer is given by *maxUF*_*LB*_(p',*R*′).

    26. Update available load for each host Є H and choose a new price strategy for every host.

    27. Repeat above steps for every incoming batch of task request.

### SGMLB algorithm

Step 1: In cloud data center, there are a large number of physical host present to serve the incoming task requests. There are situations where some hosts cannot process the requested task as it cannot meet the resource requirements of the incoming tasks. Those outlier hosts are eliminated according to Eq (1), and a new host set is formed as HS' = {(C^i^, M^i^)} whose C^i^> L_C_ and M^i^> L_M_. From the host set HS', the total resource available for each host is found out based on Eq (2). Similarly, the total resource required for each task is found using Eq (3).

Step 2: The set HS' consists of hosts with an average amount of resources. All suitable hosts for processing the task request is identified by the satisfaction factor in Eq (4). The hosts have enough resources to process the task request if the satisfaction factor is positive. The hosts have precisely the same amount of resources compared with the resource requested by the task if the satisfaction factor is zero. The host having an inadequate amount of resources to process the task negative satisfaction factor. Thus for every host and each task request, the satisfaction factor is computed, and the host with enough resource is identified. For that identified host, based on the price strategy, the follower’s utility function value is calculated as given in Eq (5).

Step 3: The followers' utility function value indicates which host can process the requested task at a cheaper price. Thus the optimal host to process the requested task is identified by the load balancer based on the follower’s utility function value given by *minUF*_*H*_(p',*R*_*j*_) Eq (6). The task is then assigned to the host with *minUF*_*H*_ and the host is made unavailable for the next task request in the batch. Thus, the host chooses its task request to process such that its resources are efficiently utilized and also the tasks are assigned to the optimal host that can be processed at a lesser price.

Step 4: Based on the task assigned to the host, the load balancer computes the utility function value for the leader based on Eqs (7) and (8). Thus, the net benefit of the leader is maximized, indicating that almost all the tasks are assigned to appropriate hosts.

Step 5: With the processing task, the host updates its available resource for the next batch of incoming tasks. Based on the availability of the resource, the price is chosen in such a way that the probability of choosing the host with the more available resource is high. Then the whole process is repeated for the next batch of incoming task requests.

The whole load balancing process of SGMLB cannot be achieved by just executing this algorithm. It is a long-term process. The optimal host for processing the requested task is found for every batch of incoming tasks request, and available resources are updated in each execution time, and the new price strategy is chosen for the host. The load balancer picks up the best response strategy hosts, thus benefitting the hosts, the task requests, and maximizing the overall performance of the load balancer.

### Stackelberg equilibrium

Equilibrium state in a game is the position where the two players’, i.e., the leader and the follower, make their decision to reach their desired outcome. At this Stackelberg equilibrium, the follower is benefitted by its minimized utility function based on the satisfaction factor and price strategy of the host. Also, the leader allocates tasks to the optimal host and maximizes its utility function based on the followers’ strategy. In the context of this problem, the Stackelberg equilibrium [31,32] is defined as the state where both the leader and follower assign and process the tasks in an optimal manner, respectively. This section explains with the set of derivatives how the Stackelberg equilibrium gets attained.

- The leader’s optimal strategy is to maximize the overall benefit by allocating tasks to every host based on the best price and computing resource availability of the hosts.
- The strategy of the host is that it processes the assigned tasks by utilizing the available computing resources. The optimal task is chosen based on the load requirement of the tasks.

The optimal strategy of the Leader and the follower is proved by Stackelberg equilibrium. By using the backward induction method, we observe that the Stackelberg equilibrium is achieved. The leader’s optimal strategy p_i_ is obtained by solving the follower’s utility function in Eq (5).

$$\frac{\partial {UF}_{H}}{\partial R_{j}}=p_{i}-\frac{w}{L_{i}}.e^{\left(1-\frac{R_{j}}{L_{i}}\right)}$$ (9)

By equating the derivative in Eq (9) to zero, we obtain the best price strategy.

$$\frac{\partial {UF}_{H}}{\partial R_{j}}=0$$ (10)

$$p_{i}=\frac{w}{L_{i}}.e^{\left(1-\frac{R_{j}}{L_{i}}\right)}$$ (11)

From Eq (11), the price range of the host is chosen as below.

Maximum price for host i is derived in Eq (12).

$$p_{i}^{max}=\max\left\{0,\frac{w}{L_{i}}.e^{\left(1-\frac{R_{j}^{min}}{L_{i}}\right)}\right\}$$ (12)

Minimum price for host i is derived in Eq (13).

$$p_{i}^{min}=\min\left\{\frac{w}{L_{i}}.e^{\left(1-\frac{R_{j}^{max}}{L_{i}}\right)},p_{CSP}\right\}$$ (13)

Note that the price p_i_ values must be less than p_CSP_, where p_CSP_ is the price from Cloud Service Provider.

$$p_{i}=\frac{w}{L_{i}}.e^{\left(1-\frac{R_{j}}{L_{i}}\right)}<p_{CSP}$$ (14)

From the leader’s price strategy, the follower’s optimal strategy is given in Eq (15).

$$R_{j}=L_{i}(1-\ln\frac{p_{i}.L_{i}}{w})$$ (15)

By taking the second-order derivative of (9) we obtain a positive value which implies the set of R_j_ is proved to be convex and so the range for R_j_ is defined to be [$R_{j}^{min},R_{j}^{max}$] in Eq (16).

$$\frac{\partial^{2}{UF}_{H}}{\partial R_{j}^{2}}=\frac{w}{{L_{i}}^{2}}.e^{\left(1-\frac{R_{j}}{L_{i}}\right)}>0$$ (16)

From Rj value the utility function of the leader can be rewritten as, mentioned in Eqs (17) and (18)

$${UF}_{LB}(p\prime ,R\prime )=\sum_{i=1}^{n}p_{i}.L_{i}(1-\ln\frac{p_{i}.L_{i}}{w})–\sum_{i=1}^{n}w.e^{1-\frac{L_{i}(1-\ln\frac{p_{i}.L_{i}}{w})}{L_{i}}}$$ (17)

$${UF}_{LB}(p\prime ,R^{\prime })=\sum_{i=1}^{n}p_{i}.L_{i}(1-\ln\frac{p_{i}.L_{i}}{w})–p_{i}.L_{i}-w$$ (18)

Solving the above function in Eq (18), the second-order derivative of the function is a negative value, which proves leaders' price strategy p' is a concave function.

$$\frac{\partial^{2}{UF}_{LB}(p',R^{\prime })}{\partial p_{i}^{2}}=-\frac{w}{p_{i}}<0$$ (19)

The weight factor w is a constant and obtained using the inequality function in Eq (16).

$$w<p_{CSP}L_{i}e^{\left(\frac{R_{j}^{max}}{L_{i}}-1\right)}$$ (20)

## Simulation environment

The cloud environment is simulated using the ‘CloudSim’ framework. The Algorithm in section 4 has been programmed in ‘CloudSim’ environment, and the simulation for the following input tasks was executed to display the performance metrics. The cloud data center is formed with a minimum of 100 physical hosts with a different available computing resource. Around 20 batches of jobs with each batch consisting of 25 to 75 tasks with varying load requirements are provided to the physical host in the simulation environment. The results are captured and documented here for 200 to 1000 tasks. The output results are captured, and visualized on varied performance metrics like makespan, failed number of tasks, throughput, resource utilization, front-end error rate, and price-load matrix. The simulations are coded in Eclipse IDE using Java on a 4.2 GHz Intel Core i7 processor with 16 GB RAM.

## Results and discussion

In this section, the proposed SGMLB approach is compared with Flow-Shop scheduling and random allocation in deploying tasks to hosts in the cloud data center. The random allocation method is followed to allocate the task as it is simple and does not require more system information. Task allocation is unbiased in such a way that no single host in data center is overloaded, and it restricts systematic errors. In Random Allocation Scheduling algorithm [33], the incoming 'm' number of tasks at time 't' is processed by 'n' number of physical hosts at random using CloudSim's inbuilt random allocation utilities. Another method considered for task allocation is Flow-Shop scheduling. The repetitive concept of Flow Shop Scheduling has many benefits like improved execution efficiency, resource utilization, reduced processing time of the tasks, and optimization of the load balancer in an easy manner. In Flow shop scheduling algorithm [34], 'm' number of tasks arriving at time t are assigned among 'n' number of physical hosts. Each batch of tasks at time ‘t’ is scheduled using Johnson’s rule, and then every task is assigned to the host at the cloud data center in a sequence. This process is repeated for all the tasks in every batch. In CloudSim environment, all three algorithms were simulated by setting the appropriate number of tasks with varying load requirements and hosts with varying computing resources. The following aspects like makespan, resource utilization, failed number of tasks, throughput, front-end error rate is compared, and price value for the available load is also graphed.

### Makespan

**Fig 3:**
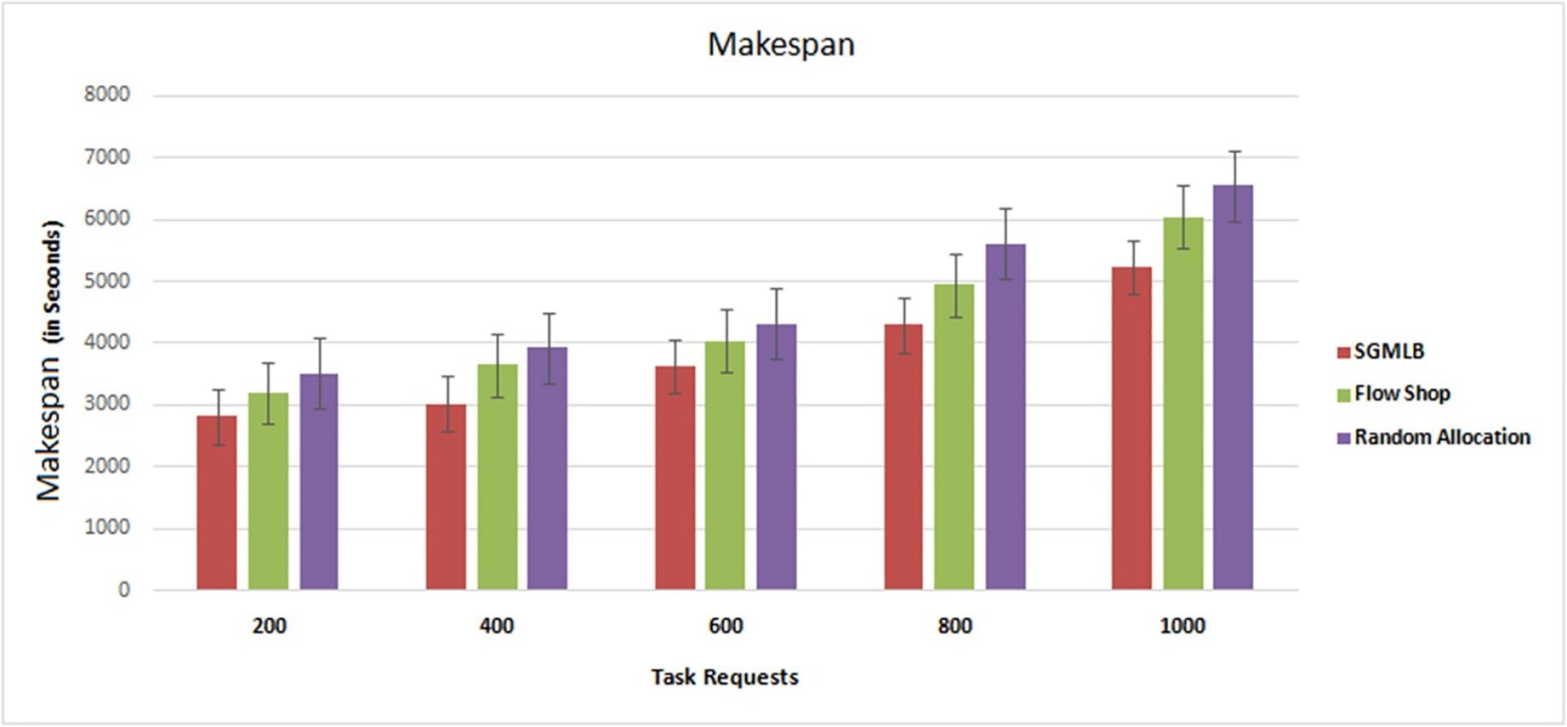
Makespan analysis.

Makespan is the total time for processing the tasks. (Fig 3) shows the result of makespan value in “Table 1” for the three models. Arbitrary deployment of the task to the physical host on the cloud data center is carried out by a random allocation model. And the flow shop scheduling finds the optimal sequence of jobs and deploys the tasks on the host on the cloud data center. While in SGMLB, the load balancer calculates the utility function of every available host and deploys the tasks on the host with the minimum utility function value. Multiple batches, each with different number of task requests, are submitted in the simulated environment of the cloud data center using ‘Random Allocation’, ‘Flow Shop scheduling’, and by using the SGMLB scheduling algorithm. With the increase in the number of incoming task requests, the time to process all submitted tasks was observed to be increasing. The makespan value for a maximum of 200 number of tasks requests is computed in the simulation environment using three different methods, and in general, the makespan value is found to increase with the increase in the number of tasks in all the three approaches. When compared with random allocation and flow-shop scheduling, SGMLB methods have relatively lower makespan value as it uses optimal hosts to deploy the submitted tasks request in the data center.

**Table 1:** Makespan values in seconds for different task requests.

	Makespan (in Seconds)		
Task Requests	SGMLB	Flow Shop	Random Allocation
200	2820	3200	3520
400	3020	3650	3930
600	3625	4045	4325
800	4300	4950	5615
1000	5330	6050	6555

### Failed no of tasks

**Fig 4:**
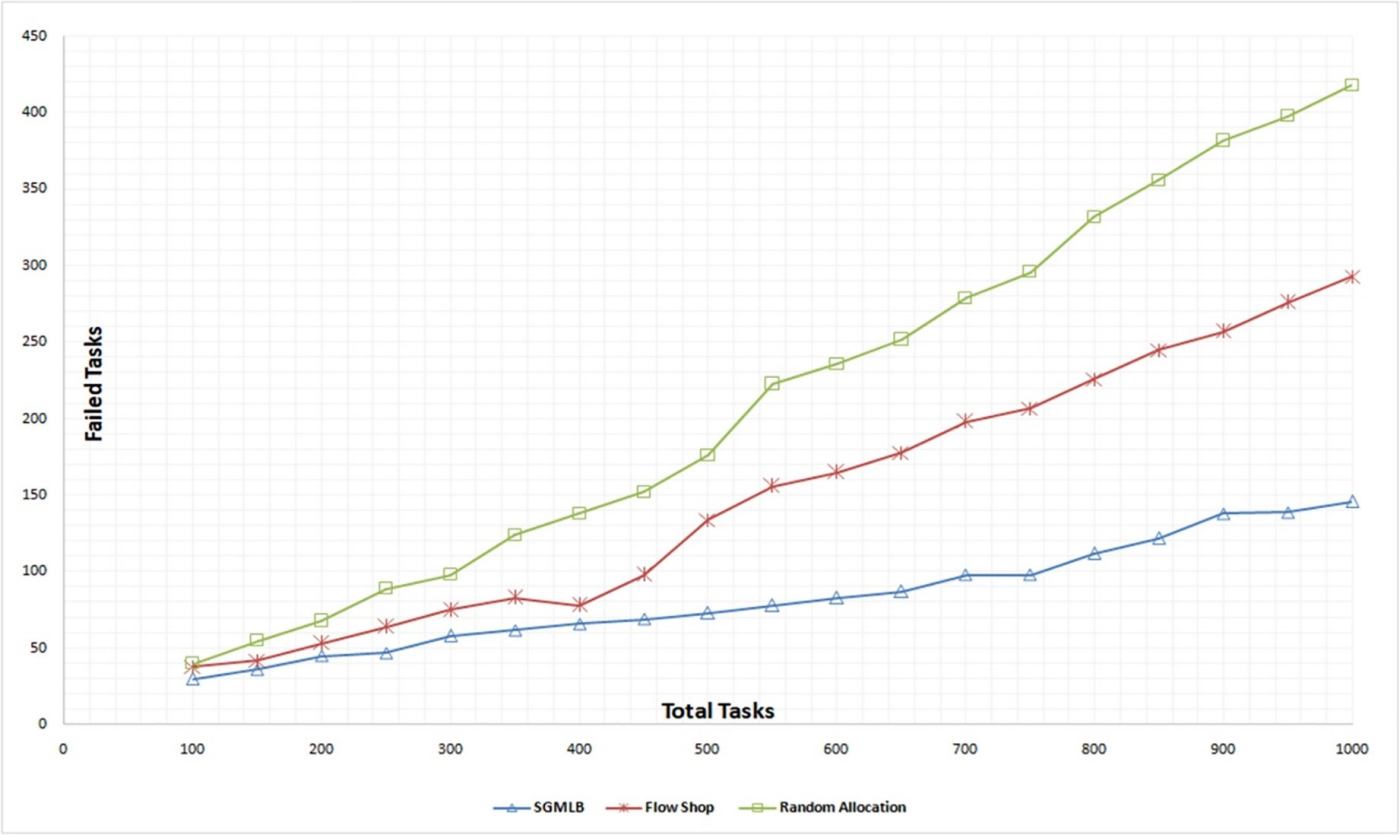
Failed number of tasks analysis.

Allotted tasks can fail due to host failure or when the tasks are allocated to hosts with an insufficient amount of resources. These types of failures are simulated in cloudsim. In random allocation method, the tasks are allocated at random to the host, while in flow-shop scheduling, the tasks are allotted in sequence. SGMLB utilizes the satisfaction factor to identify the appropriate host set and select the optimal host to deploy the task request. The simulation results show that the SGMLB algorithm efficiently deploys the tasks to the host with a sufficient amount of resources. The results of the SGMLB algorithm, flow-shop, and random allocation is tabulated in “Table 2,” and the results are compared in(Fig 4), which shows that the number of tasks failed has reduced when compared to flow shop scheduling and random allocation methods.

**Table 2:** Number of tasks failed against total tasks submitted.

	Failed No of Tasks		
Total Tasks	SGMLB	Flow Shop	Random Allocation
200	45	53	68
400	66	78	138
600	83	165	236
800	112	245	356
1000	146	293	418

### Throughput

**Fig 5:**
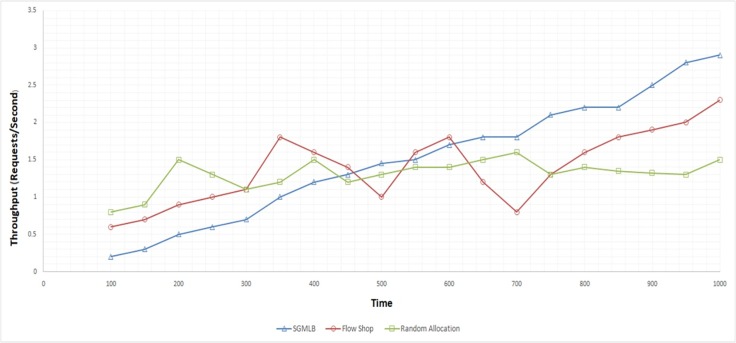
Throughput analysis.

Throughput is a measure of the number of tasks gets completed in a given period. The simulation in the Cloudsim environment measured the performance of the SGMLB algorithm in deploying the tasks request per second in the cloud data center. As per the proposed algorithm in this paper, the satisfaction factor and utility functions with the price strategy played a pivotal role in choosing the optimal host for deploying the incoming tasks. The number of tasks getting allocated to optimal hosts and completed in a given time frame is observed to have increased in the SGMLB method, whereas the throughput of random allocation and flow-shop scheduling method was observed to be very less. The throughput value is tabulated in “Table 3” for the three approaches. The performance of the SGMLB algorithm in (Fig 5) was found to be minimal compared to the other two approaches at the beginning but has improved gradually over time, as observed in the graph below. The performance of the other two approaches is inconsistent with drastic increase and decrease in the throughput values, which is not the desired KPI (Key Performance indicator) in a cloud-based IaaS.

**Table 3:** Throughput value for task requests per second.

	Throughput (Requests/Second)		
Task Requests	SGMLB	Flow Shop	Random Allocation
200	0.5	0.9	1.5
400	1.2	1.6	1.5
600	1.7	1.8	1.4
800	2.2	1.8	1.35
1000	2.9	2.3	1.5

### Resource utilization

**Fig 6:**
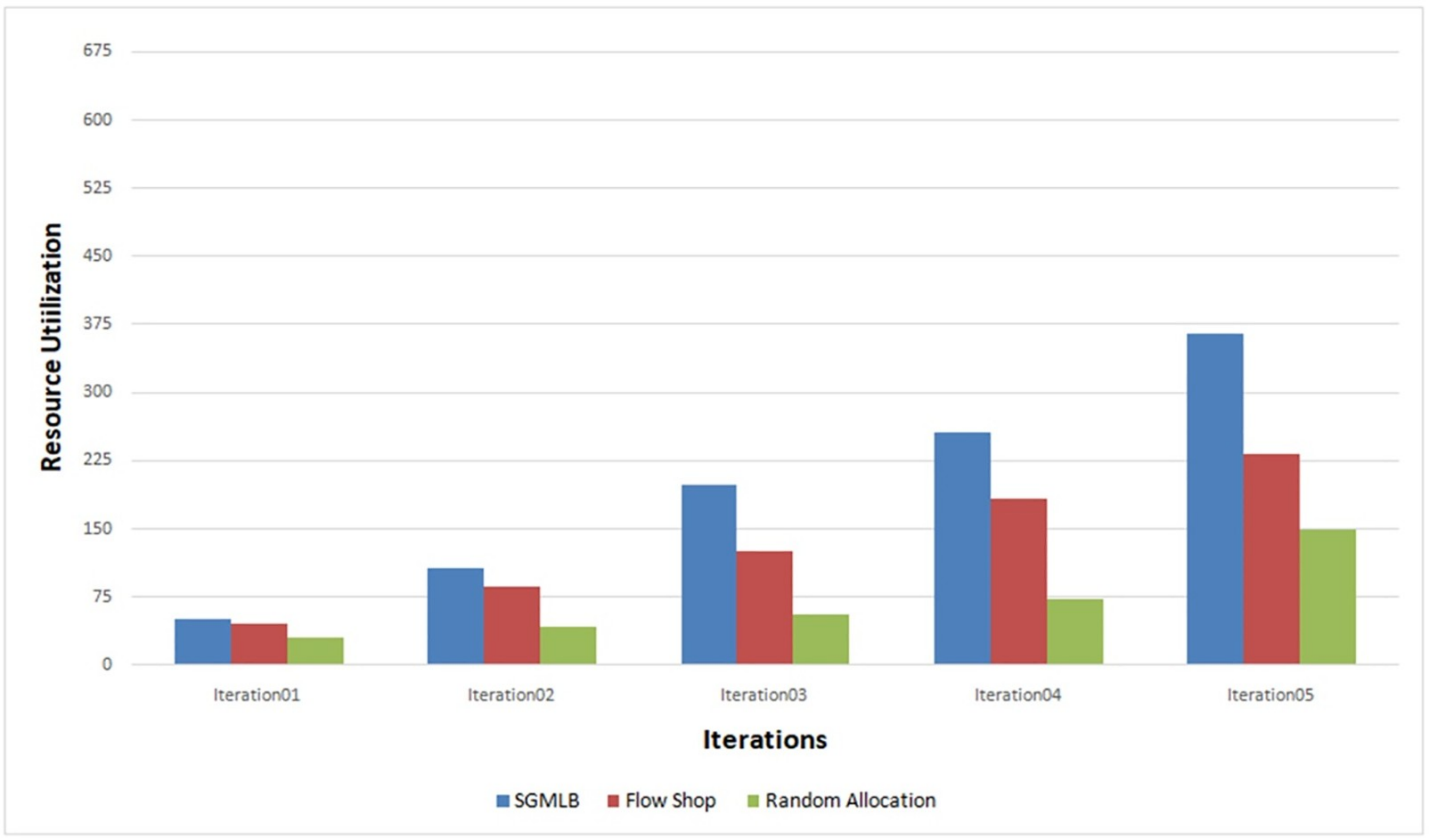
Resource utilization analysis.

This measure indicates how the computing resources of the hosts are efficiently utilized. In random allocation and flow-shop scheduling, the tasks being scheduled to a particular host without considering the hosts’ maximum or minimum load availability. Where-as in the SGMLB model, the host, gets allotted with the right job for processing, i.e., the host with more resource is being allocated first, then followed by the next host, which results in efficient resource utilization compared with random allocation and flow-shop scheduling. The experimental result of SGMLB, Flow-Shop scheduling, Random allocation have been tabulated in “Table 4,” and the result shows that the SGMLB allocates the tasks to a host that has optimal computing resources based on the follower’s utility function as explained in the proposed algorithm. The proposed model ensures that the computing resources are efficiently utilized that is shown in (Fig 6).

**Table 4:** Resource utilization in every iteration.

	Resource Utilization		
Iteration	SGMLB	Flow Shop	Random Allocation
Iteration01	50	45	30
Iteration02	107	87	43
Iteration03	198	126	56
Iteration04	257	183	72
Iteration05	365	232	150

### Front-end error rate

**Fig 7:**
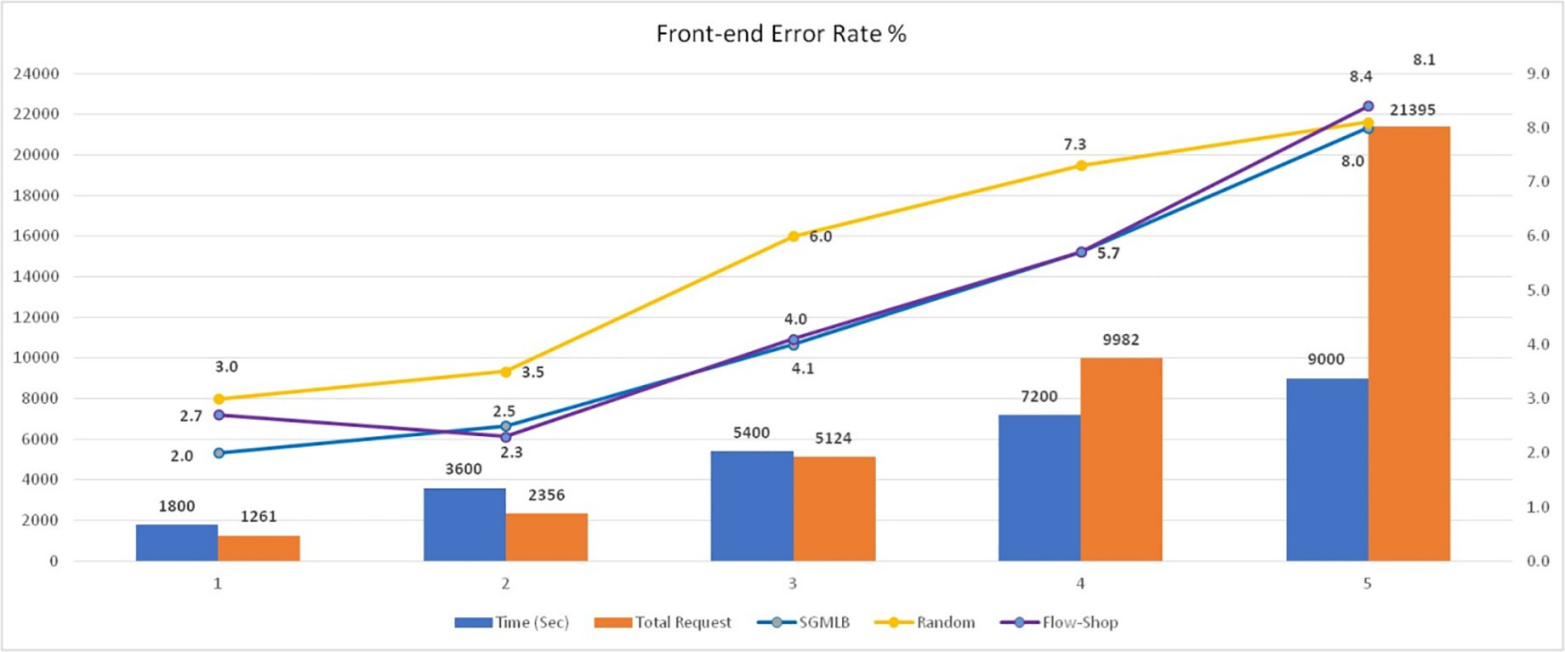
Front-end error rate analysis.

Front-end error rate measured in the context of this experiment is defined as the percentage of error that the load balancer returns to the calling client when it fails to identify a host in the data center to allocate a given task. Front-end error rate is tabulated in "Table 5" and (Fig 7) shows the results for the three models. The continuous stream of requests was submitted for a pre-defined period as part of this experiment instead of submitting the requests in small batches, and the front-end error rate is measured for that simulation period. The requests were submitted for a period of 30 minutes to 150 minutes, and the front-end error rate was measured. Results show that the 'SGLMB' algorithm has a slight advantage over ‘Random Allocation’ but observed to perform on par with ‘Flow Shop scheduling.

**Table 5:** Front-end error rate.

Time (Sec)	Total Request	Front-end Error Rate %		
		SGMLB	Random	Flow-Shop
1800	1261	2	3	2.7
3600	2356	2.5	3.5	2.3
5400	5124	4	6	4.1
7200	9982	5.7	7.3	5.7
9000	21395	8	8.1	8.4

### Price of load capacity

Price of the host is the cost at which the host lends its computing resource for the upcoming jobs for processing. This price value in the cloud data center is chosen based on hosts—available computing resource. The price is set to a higher value for the host if its available computing resources are less so that the probability of this host getting chosen for task processing is very less.

**Fig 8:**
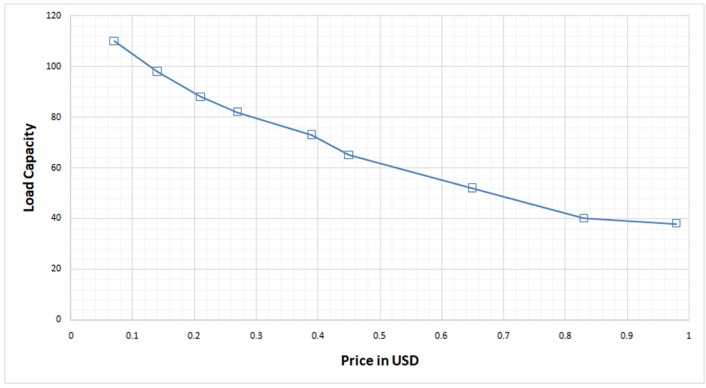
Price value computed for load capacity.

Similarly, the price is set to a lower value for the host if its available computing resource is sufficient enough to process an incoming task so that the probability of this host getting chosen for task processing is high. So, the price for every host in the cloud data center is set based on the maximum and minimum load request it can able to process, which has also been shown in Stackelberg equilibrium. This price strategy for available load capacity has been shown in “Table 6”. (Fig 8) shows the price value in USD for different available load capacity.

**Table 6:** Price strategy for the hosts based on available load.

Load Capacity	Price ($)
40	0.83
65	0.45
82	0.27
98	0.14
110	0.07

## Conclusion and future work

This paper has proposed the deployment of tasks in a load-balanced way using SGMLB game-theoretical approach reinforced by the satisfaction factor for aggregating the optimal hosts. The definite hosts are filtered, and the leader-follower strategy has been employed to identify optimal hosts with their available resources. Then, SGMLB has Stackelberg game mode that allocates tasks to suitable hosts based on the resource requirement, price strategy of the hosts, and the available resources on the host benefitting the hosts, the task requests, and maximizes the overall performance of the load balancer. The simulations results have shown (i) that the tasks are deployed at a faster rate to the hosts in the data center, (ii) reduced number of failed tasks due to effective resource utilization, (iii) Increased throughput and makespan values. The proposed SGMLB model is observed to have utilized the resources 60% more effectively as compared to the flow-shop scheduling and random-allocation model. The proposed SGMLB model balances the load incurred by the task request in the long-term perspective.

In this way, SGMLB can choose an optimal host from the aggregated set of hosts based on a leader-follower game-theoretical strategy to deploy tasks by adding advantage to the hosts and the tasks. Latency in load balancing is one of the critical metric to be considered while allocating tasks. The leader, which is the load balancer as per the proposed SGMLB algorithm, must also address latency as one of its metrics while allocating tasks. The future work will consider the latency as one of the parameters and optimize the solution. The proposed model can also be extended to load balancing jobs in real-time streaming analytics in a cloud-based big data platform.

## Supporting information

S1 Dataset (XLSX)

S2 Dataset (XLSX)

S3 Dataset (XLSX)

S4 Dataset (XLSX)

S5 Dataset (PDF)

## References

[1] Calheiros R. N., Ranjan R., Beloglazov A., De Rose C. A., &Buyya R. (2011). CloudSim: a toolkit for modeling and simulation of cloud computing environments and evaluation of resource provisioning algorithms. *Software*: *Practice and experience*, 41(1), 23–50.

[2] Garg S. K., Versteeg S., &Buyya R. (2013). A framework for ranking of cloud computing services. *Future Generation Computer Systems*, 29(4), 1012–1023.

[3] Santra, S., & Mali, K. (2015, September). A new approach to survey on load balancing in VM in cloud computing: Using CloudSim. In 2015 International Conference on Computer, Communication and Control (IC4) (pp. 1–5). IEEE.

[4] Mazrekaj A., Shabani I., &Sejdiu B. (2016). Pricing schemes in cloud computing: an overview. *International Journal of Advanced Computer Science and Applications*, 7(2), 80–86.

[5] Wu, L., Garg, S. K., &Buyya, R. (2011, May). SLA-based resource allocation for software as a service provider (SaaS) in cloud computing environments. In *Proceedings of the 2011 11th IEEE/ACM International Symposium on Cluster, Cloud and Grid Computing* (pp. 195–204). IEEE Computer Society.

[6] Nayyar A. (2019). *Handbook of Cloud Computing*: *Basic to Advance research on the concepts and design of Cloud Computing*. BPB Publications.

[7] Rastogi, G., & Sushil, R. (2015, October). Analytical literature survey on existing load balancing schemes in cloud computing. In *2015 International Conference on Green Computing and Internet of Things (ICGCIoT)* (pp. 1506–1510). IEEE.

[8] Aslam, S., & Shah, M. A. (2015, December). Load balancing algorithms in cloud computing: A survey of modern techniques. In *2015 National Software Engineering Conference (NSEC)* (pp. 30–35). IEEE.

[9] Tang F., Yang L. T., Tang C., Li J., & Guo M. (2016). A dynamical and load-balanced flow scheduling approach for big data centers in clouds. *IEEE Transactions on Cloud Computing*, 6(4), 915–928.

[10] Babu K. R., & Samuel P. (2016). Enhanced bee colony algorithm for efficient load balancing and scheduling in cloud In *Innovations in bio-inspired computing and applications* (pp. 67–78). Springer, Cham.

[11] Farrag, A. A. S., Mahmoud, S. A., & El Sayed, M. (2015, December). Intelligent cloud algorithms for load balancing problems: A survey. In *2015 IEEE Seventh International Conference on Intelligent Computing and Information Systems (ICICIS)* (pp. 210–216). IEEE.

[12] Kaur A., Gupta P., Singh M., & Nayyar A. (2019). Data placement in era of cloud computing: a survey, taxonomy and open research issues. *Scalable Computing: Practice and Experience*, 20(2), 377–398.

[13] Deep G., Mohana R., Nayyar A., Sanjeevikumar P., & Hossain E. (2019). Authentication Protocol for Cloud Databases Using Blockchain Mechanism. *Sensors*, 19(20), 4444. 10.3390/s19204444 PMC6832710 31615014

[14] Zhao J., Yang K., Wei X., Ding Y., Hu L., & Xu G. (2015). A heuristic clustering-based task deployment approach for load balancing using Bayes theorem in cloud environment. *IEEE Transactions on Parallel and Distributed Systems*, 27(2), 305–316.

[15] Wang Y., Tao X., He Q., &Kuang Y. (2016). A dynamic load balancing method of cloud-center based on SDN. *China Communications*, 13(2), 130–137.

[16] Somula R., &Sasikala R. (2018). Round robin with load degree: An algorithm for optimal cloudlet discovery in mobile cloud computing. Scalable Computing: Practice and *Experience*, 19(1), 39–52.

[17] Patel S., & Bhatt M. (2017). Implementation of Load balancing in Cloud computing through Round Robin & Priority using cloudSim. International Journal for Rapid Research in Engineering Technology & Applied *Science*, 3(11).

[18] Zhang W., Han S., He H., & Chen H. (2017). Network-aware virtual machine migration in an overcommitted cloud. *Future Generation Computer Systems*, 76, 428–442.

[19] Sun Z., Huang X., & Ma Y. (2008). Load Balancing Strategies to Solve Flowshop Scheduling on Parallel Computing. *arXiv preprint arXiv*:*0809*.*3285*.

[20] Wooldridge M. (2012). Does game theory work? *IEEE Intelligent Systems*, 27(6), 76–80.

[21] Tripathi R., Vignesh S., Tamarapalli V., Chronopoulos A. T., &Siar H. (2017). Non-cooperative power and latency aware load balancing in distributed data centers. *Journal of Parallel and Distributed Computing*, 107, 76–86.

[22] Xiao Z., Tong Z., Li K., & Li K. (2017). Learning non-cooperative game for load balancing under self-interested distributed environment. *Applied Soft Computing*, 52, 376–386.

[23] Fernández-Cerero D., Fernández-Montes A., Jakobik A., &Kolodziej J. (2018). Stackelberg Game-Based Models In Energy-Aware Cloud Scheduling. In *ECMS* (pp. 460–467).

[24] Song S., Lv T., & Chen X. (2014). Load balancing for future internet: an approach based on game theory. *Journal of Applied Mathematics*, 2014.

[25] Nan G., Mao Z., Yu M., Li M., Wang H., & Zhang Y. (2013). Stackelberg game for bandwidth allocation in cloud-based wireless live-streaming social networks. *IEEE Systems Journal*, 8(1), 256–267.

[26] Yu M., & Hong S. H. (2015). A real-time demand-response algorithm for smart grids: A stackelberg game approach. *IEEE Transactions on Smart Grid*, 7(2), 879–888.

[27] Wang X., Chen X., Wu W., An N., & Wang L. (2015). Cooperative application execution in mobile cloud computing: A Stackelberg game approach. *IEEE Communications Letters*, 20(5), 946–949.

[28] Duong N. D., Madhukumar A. S., &Niyato D. (2015). Stackelberg Bayesian game for power allocation in two-tier networks. *IEEE Transactions on Vehicular Technology*, 65(4), 2341–2354.

[29] Tran N. H., Tran D. H., Ren S., Han Z., Huh E. N., & Hong C. S. (2015). How geo-distributed data centers do demand response: A game-theoretic approach. *IEEE Transactions on Smart Grid*, 7(2), 937–947.

[30] Wang H., Huang J., Lin X., & Mohsenian-Rad H. (2015). Proactive demand response for data centers: A win-win solution. *IEEE Transactions on Smart Grid*, 7(3), 1584–1596.

[31] d'Aspremont C., & Gérard-Varet L. A. (1980). Stackelberg-solvable games and pre-play communication. *Journal of Economic Theory*, 23(2), 201–217.

[32] Roughgarden T. (2004). Stackelberg scheduling strategies. *SIAM journal on computing*, 33(2), 332–350.

[33] Shah S. C., Chauhdary S. H., Bashir A. K., & Park M. S. (2010). A centralized location-based job scheduling algorithm for inter-dependent jobs in mobile ad hoc computational grids. *Journal of Applied Sciences*, 10(3), 174–181.

[34] Kuo I. H., Horng S. J., Kao T. W., Lin T. L., Lee C. L., Terano T., & Pan Y. (2009). An efficient flow-shop scheduling algorithm based on a hybrid particle swarm optimization model. *Expert systems with applications*, 36(3), 7027–7032.

