# Supplementary material for: Game theoretical approach for load balancing using SGMLB model in cloud environment
Source: PLoS One. 2020 Apr 20;15(4):e0231708. doi: 10.1371/journal.pone.0231708 (PMC7170225; doi:10.1371/journal.pone.0231708)
Supplement: S5 Dataset — (PDF) [file pone.0231708.s005.pdf]

SGMLB – Cloudsim Simulation – Dataset Attributes Explanation

This Document gives a high-level description of the data set used for cloudsim simulation as part of SGMLB model implementation and comparison with other methods.

- The Dataset consists data for 5 iterations for each of the measurements namely Makespan, Count of Failed Tasks, Throughput and Resource Utilization.
- Each Excel workbook contains data for each of the measurements namely Makespan, Count of Failed Tasks, Throughput and Resource Utilization.
- Data for each iteration is given in 5 separate sheets in the workbook.

Following are important fields in the data set:

- **Timestamp [ms in epoch]** - Time in epoch time format at millisecond granularity. Denotes the time at which job was submitted. This value was not used by the simulation code. This is just for logging and track the job status.
- **BatchID** - Denotes the Unique ID assigned to a batch in which a task was submitted
- **Iteration** - Denotes the iteration number
- **RequestId** - Denotes the unique identifier of the task submitted
- **Task Load** - Indicates the actual task executed in each of the node in the simulation environment. The number indicates the count of for loop used to iteratively encrypt a static string using AES 256 encryption algorithm.
- **CPU Speed** - Denotes the CPU Speed of the virtual host in simulated environment. This is kept as a constant value throughout the simulation experiment.

For the below list of CPU, Cores and Memory parameters only the max value was used in this simulation experiment. The dataset includes the minimum value for these parameters. However, the simulation was implemented to ignore these minimum values.

- **CPU Requested [Min]** - Denotes the minimum CPU requested in the simulated virtual host. This was not used in this experiment but included in dataset for future work
- **CPU Requested [Max]** - Denotes the maximum CPU requested in the simulated virtual host.
- **Cores Requested [Min]** - Denotes the minimum CPU cores requested in the simulated virtual host. This was not used in this experiment but included in dataset for future work
- **Cores Requested [Max]** - Denotes the maximum CPU cores requested in the simulated virtual host.
- **Memory Requested (GB) [Min]** - Denotes the minimum memory to be allotted to the simulated virtual host. This was not used in this experiment but included in dataset for future work
- **Memory Requested (GB) [Max]** - Denotes the maximum memory to be allotted to the simulated virtual host.
